# Supplementary material for: BAD-LAMP controls TLR9 trafficking and signalling in human plasmacytoid dendritic cells
Source: Nat Commun. 2017 Oct 13;8:913. doi: 10.1038/s41467-017-00695-1 (PMC5640662; doi:10.1038/s41467-017-00695-1)
Supplement: Supplementary file 1 — Supplementary information [file 41467_2017_695_MOESM1_ESM.pdf]

Description of Supplementary Files

File Name: Supplementary Information

Description: Supplementary figures, supplementary tables

File Name: Peer Review File

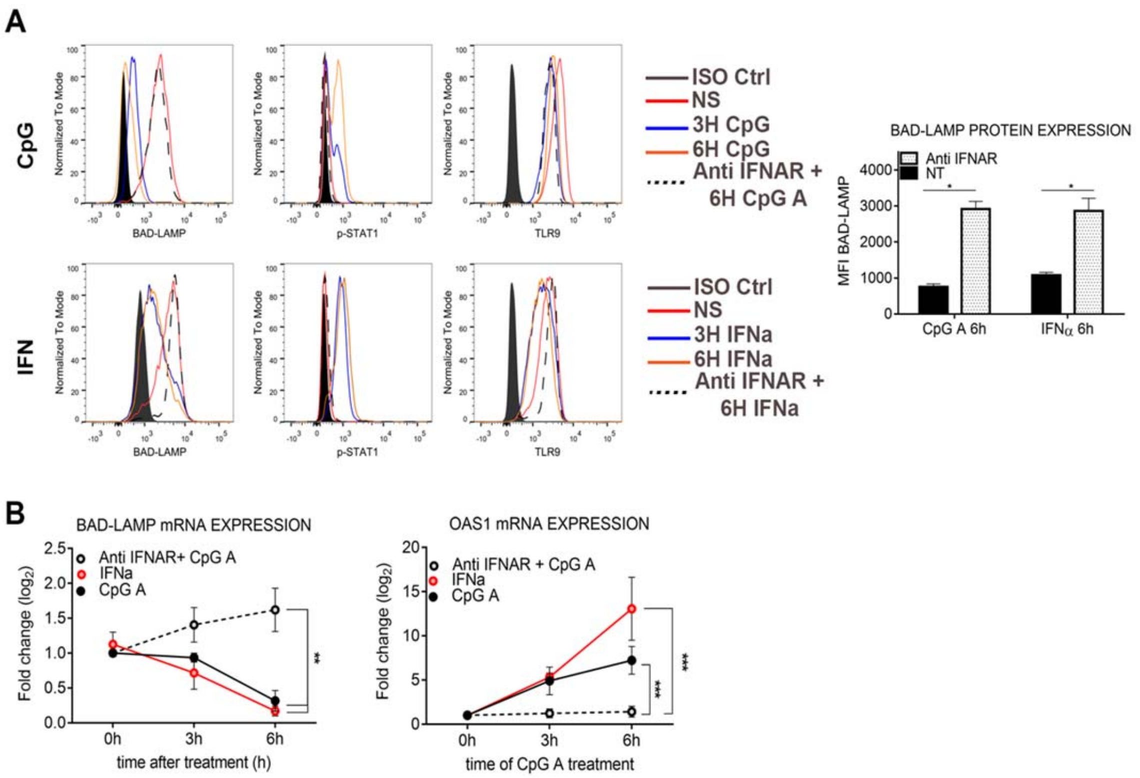

Supplementary Figure 1: Exposure to IFN down-modulates BAD-LAMP levels.

A. *Left)* Histogram plots from FACS intracellular staining of BAD-LAMP, phosphorylated-STAT1 and TLR9 in CAL-1 at steady state (red line), 3h (blue line), 6h (orange line) after CpG A (upper row) or IFN α (bottom row) stimulations. Pre-treatment for 1h with anti-IFNAR blocking antibody was used to block IFN signaling (dashed line). Full grey histogram represents isotype control. Data are representative for 3 independent experiments minimum. *Right)* Levels of BAD-LAMP as MFI from FACS intracellular staining on CAL-1 stimulated with CpG A or IFN-α for 6h with (dashed) or without (black) IFNAR blocking antibodies. Data +/- SD from 3 independent experiments. B. BAD-LAMP (left) and OAS1 (ISG. right) mRNA expression in CAL-1 stimulated with CpG A (black), IFN-α (red) and CpG A + anti-IFNAR antibody pretreatment (dashed) for indicated times. Raw data have been normalized to housekeeping gene (GAPDH) and graphic represents fold change +/- SD compared to non-stimulated cells from 3 independent experiments.

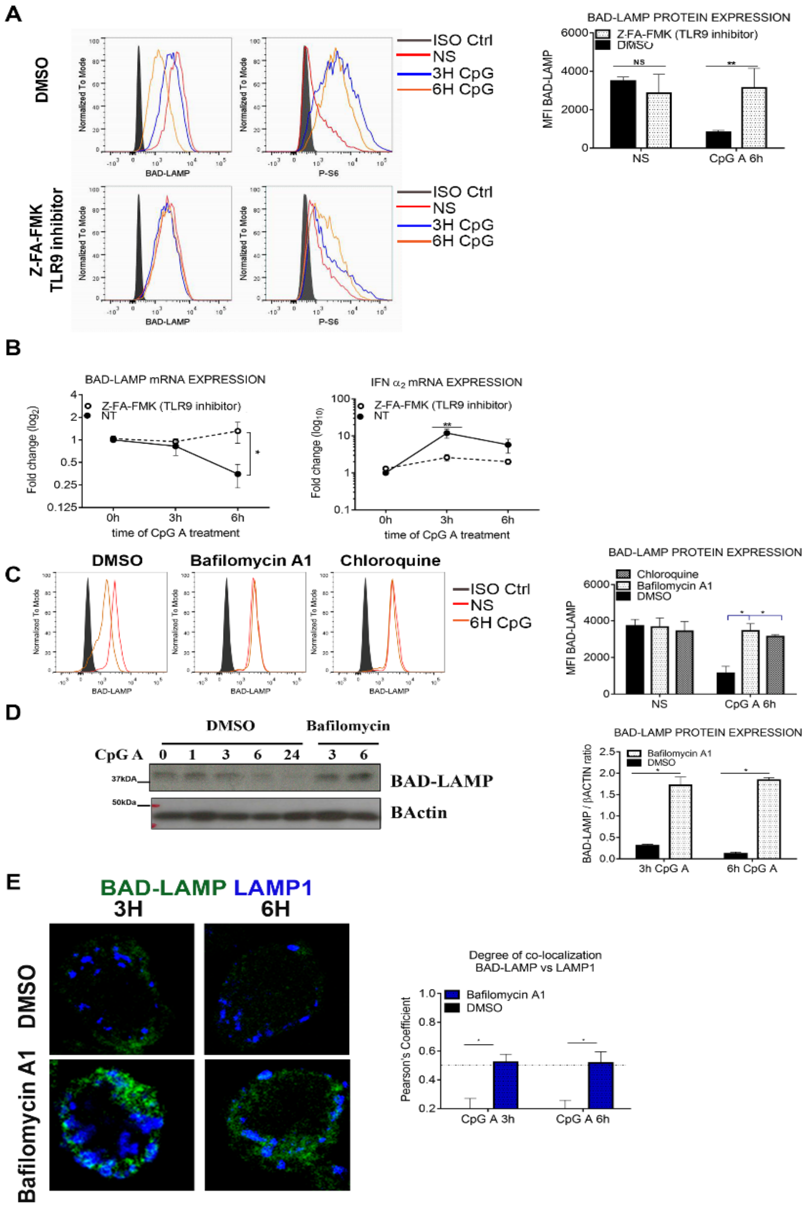

Supplementary Figure 2: TLR9 inhibition and high endosomal pH abrogates BAD-LAMP down-modulation

A. *Left)* Histogram plots from FACS intracellular staining of BAD-LAMP and phosphorylated form of S6 ribosomal protein (used activation control) in CAL-1 at steady state (red line), 3h (blue line), 6h (orange line) after CpG A stimulation. Pre-treatment for 4h with protease inhibitor ZA-FA-FMK was used to block TLR9 signaling (bottom row). Full grey histogram represents isotype control. Data are representative for 3 independent experiments. *Right)* Levels of BAD-LAMP as MFI from FACS intracellular staining on CAL-1 at steady state or treated with CpG A 6h with (dashed) or without (black) ZA-FA-FMK pretreatment. Data +/- SD from 3 independent experiments.B. BAD-LAMP (left) and IFN□$_2$ (right) mRNA expression in CAL-1 stimulated for indicated times with CpG A (black) or ZA-FA-FMK pretreatment (dashed). Raw data have been normalized to housekeeping gene (GAPDH) and graphics represent fold change +/- SD compared to non-stimulated cells from 3 independent experiments.C. Left) Histogram plots from FACS intracellular staining of BAD-LAMP in CAL-1 at steady state (red line) and 6h (orange line) after CpG A stimulation. Treatment with Bafilomycin A1 and Chloroquine was used to block Endosomal acidification. Full grey histogram represents isotype control. Data are representative for 2 independent experiments. *Right)* Levels of BAD-LAMP as MFI from FACS intracellular staining on CAL-1 at steady state or treated with CpG A 6h and Bafilomycin A1 (dashed), Chloroquine (Grey) or without any additional treatment (black). Data +/- SD from 2 independent experiments.D. (Left) CAL-1 cells were treated with CpG A for indicated times with or without additional bafilomycin A1 treatment prior lysis and SDS PAGE treatment. Expression of BAD-LAMP was detected by immunoblot. Beta-actin is shown as loading control. (*Right)* Quantification of IRF7 (red) and p65 (blue) phosphorylation levels. E. Left) ICM on CAL-1 cells treated or not with Bafilomycin A1 during indicated time of CpGA treatment. Imaging of BAD-LAMP and LAMP1 have been performed. *Right)* Quantification of BAD-LAMP protein expression by Image J pixel quantification. Graphics represents data normalized to Beta actin levels +/- SD from 2 independent experiments. * $P < 0.05$ by unpaired Student's T test.

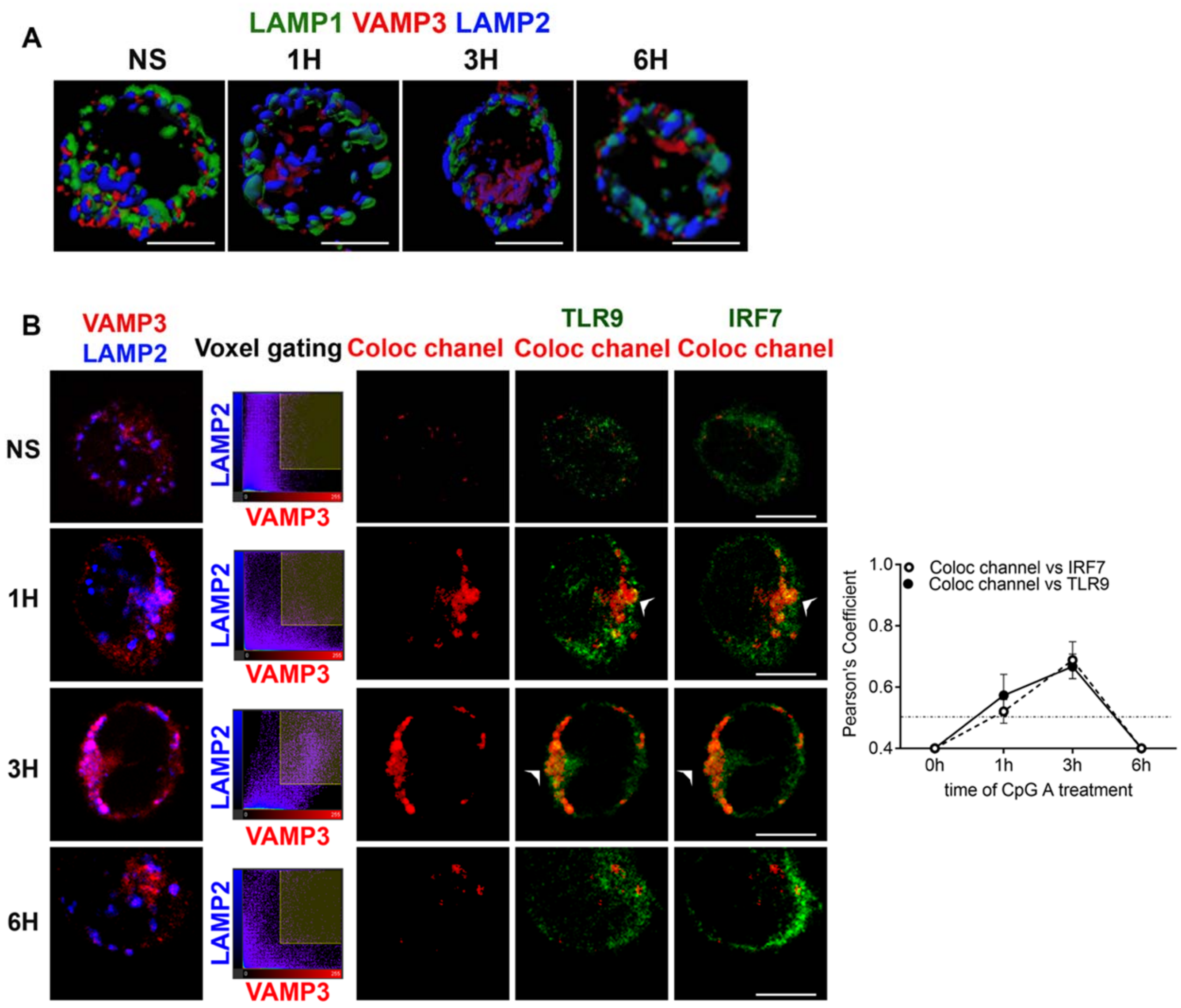

Supplementary Figure 3: TLR9 and IRF7 are targeted to a VAMP3$^+$/LAMP2$^+$ endocytic compartment.

A. Immunofluorescence confocal microscopy of CAL-1 stimulated with CpG A for indicated times. Imaging for LAMP1, LAMP2 and VAMP3 was performed. Set of several images were acquired by Z stack follow by a deconvolution processing in order to perform a 3D reconstruction with Imaris Software. Picture are representative of of at least 3 independent experiments. Scale bars = 5µm. B .Left) Imaris voxel gating analysis of immunofluorescence confocal images of CAL-1 cells treated with CpG A for indicated times. Voxel gating was performed on LAMP2 (Blue) and VAMP3 (Red) channels to generate a “Coloc channel” picture only showing VAMP3$^+$/LAMP2$^+$ compartment. This new “Coloc channel” was then merged with single TLR9 and IRF7 staining to obtained simplified tri-localization images, revealing that both TLR9 and IRF7 co-localize with VAMP3$^+$/LAMP2$^+$ compartment after of CpG A exposure. Arrowheads indicate co-localization area. Scale bars = 5µm. (Right) Quantification of the co-localisation between VAMP3$^+$/LAMP2$^+$ compartment (coloc channel) and TLR9 (black line) or IRF7 (dashed line) after CpG A stimulation was performed by Pearson’s coefficient measurement using ImageJ. Graphics represent Pearson’s coefficient means of 25 different cells +/- SD for each time point from at least 3 independent experiments. * P < 0.05 by unpaired Student’s T test.

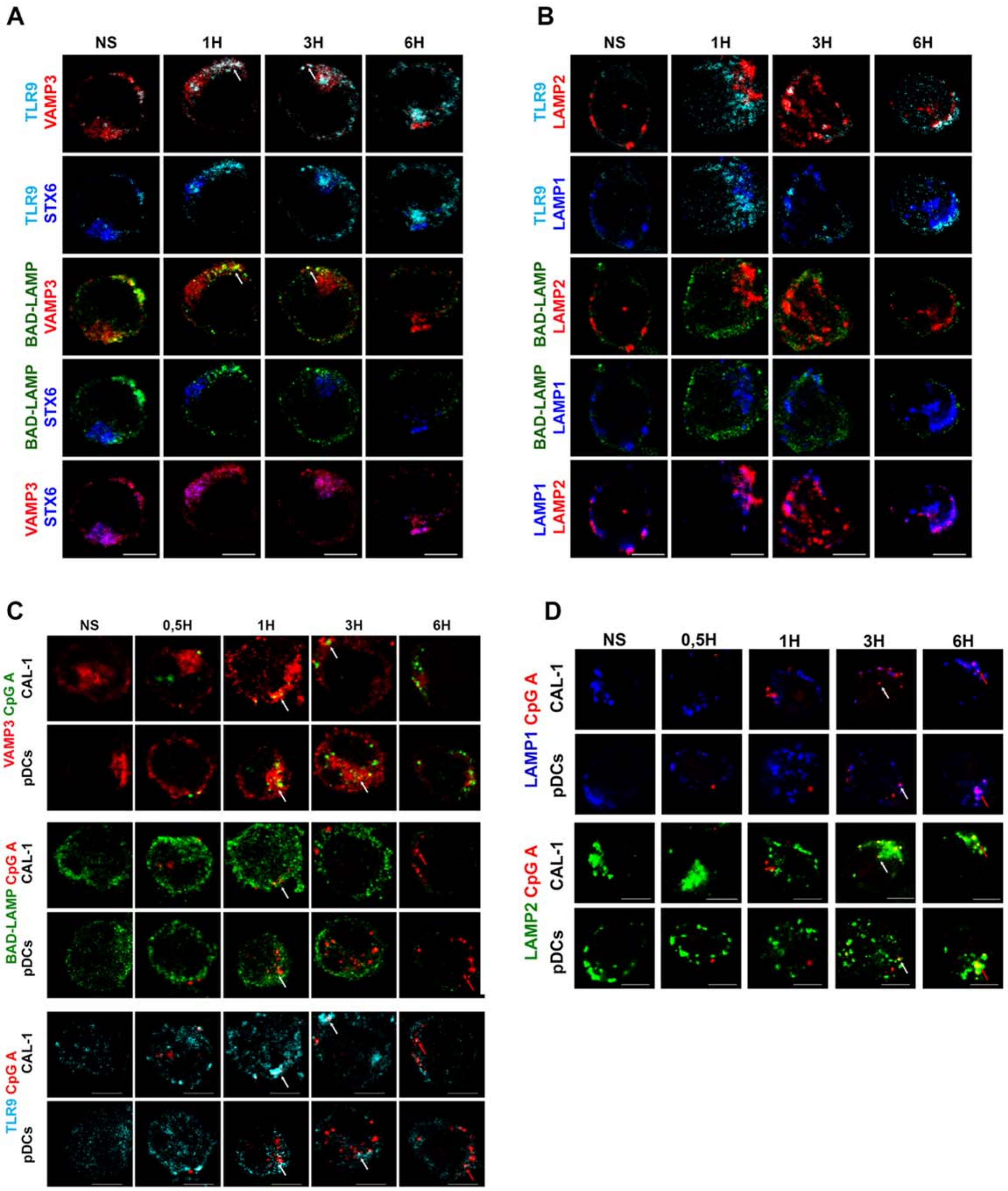

Supplementary Figure 4: TLR9 and BAD-LAMP traffic through a common endocytic route with CpG A.

A- Immunofluorescence confocal microscopy of CAL-1 stimulated with CpG A for indicated times. Imaging for TLR9, VAMP3 and Syntaxin 6 (STX6) was performed. Images are representative of at least 3 independent experiments. Arrowheads indicate co-localization area. Scale bars = 5µm. B. Immunofluorescence confocal microscopy of CAL-1 stimulated with CpG A for indicated times. Images for BAD-LAMP, TLR9, LAMP1 and LAMP2 localization are shown. Pictures are representative of at least 3 independent experiments. White arrows identify co-localization area. Scale bars = 5µm. C-D. Immunofluorescence confocal microscopy of CAL-1 treated with fluorescent CpG A for indicated times. Imaging for BAD-LAMP, VAMP3, TLR9 and CpG A (C) or LAMP1, LAMP2 and CpG A (D) was performed. Images are representative of at least 3 independent experiments. Arrows indicate co-localization area. Scale bars = 5µm. * P < 0.05 by unpaired Student's T test.

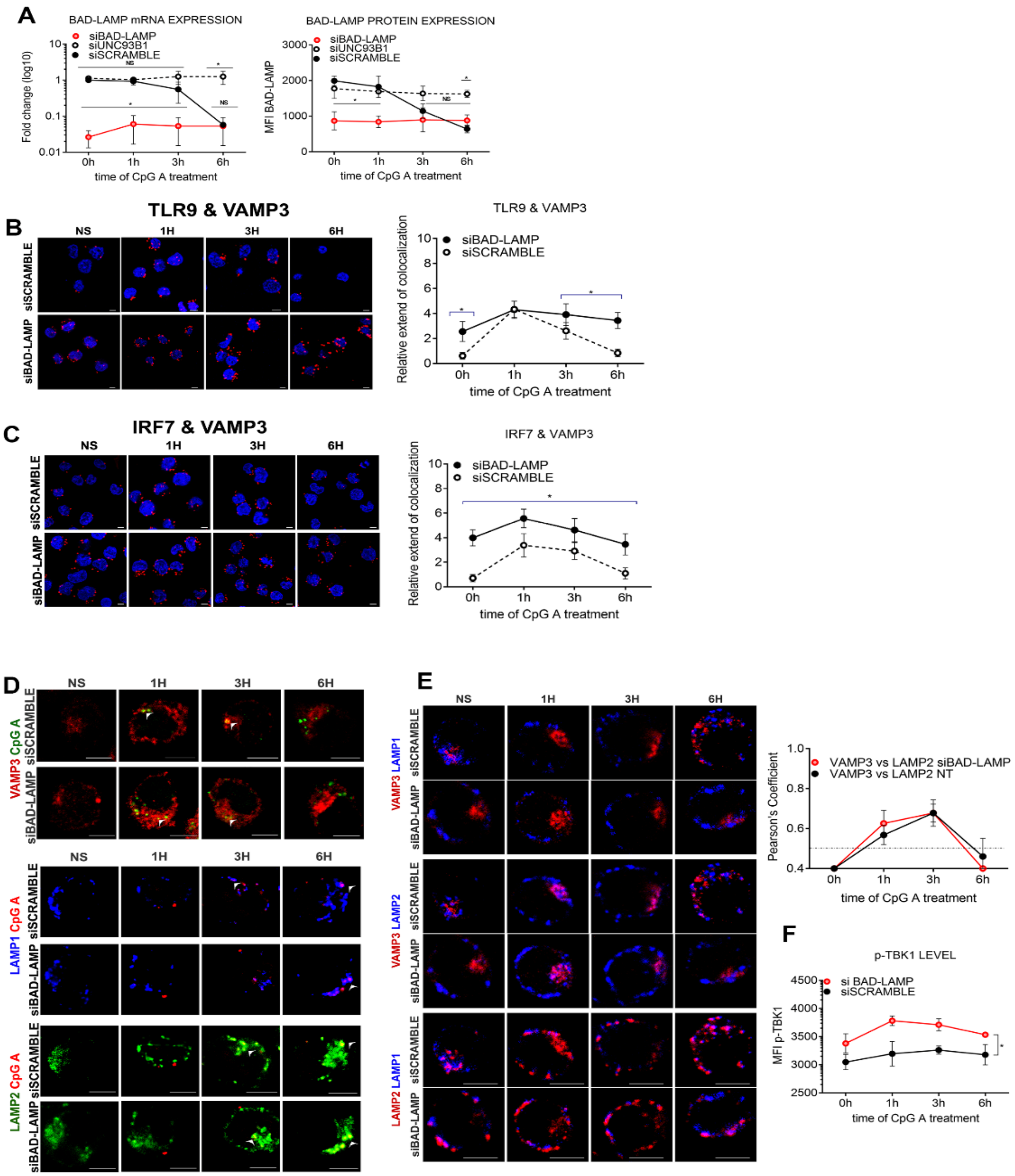

Supplementary Figure 5: BAD-LAMP silencing leads to IRF7 accumulation on TLR9 containing VAMP3 endosomes.

A. CAL-1 cells were electroporated with different siRNA (siSCRAMBLE, siBAD-LAMP and siUNC93B1) for 24hr before stimulation for indicated times with CpG. BAD-LAMP mRNA level was monitored by RT-QPCR. Raw data have been normalized to housekeeping gene (GAPDH) and graphic represents fold change +/- SD compared to non-stimulated cells from 3 independent experiments minimum. BAD-LAMP protein levels as MFI were monitored by intracellular FACS staining. Graphic represents means of MFI +/- SD from at least 3 independent experiments. B, C. (Left) Immunofluorescence proximity ligation assay (iPLA) on CAL-1 treated with control (scramble) siRNA or BAD-LAMP siRNA and then stimulated with CpG A for indicated times. Imaging TLR9 and VAMP3 (B), IRF7 and VAMP3 (C) are presented. Confocal images are representative of 2 independent experiments where the nucleus is stained by DAPI (blue) and proximity between proteins of interest is revealed by incorporation of labelled nucleotide (red). Scale bars = 5µm. (Right) Quantification of iPLA by counting the number of red dots normalized to nucleus numbers using imageJ.

Graphics represent means normalized value of 100 different cells +/- SD from n>=3 independent experiments (dots/cell). D. Immunofluorescence confocal microscopy of CAL-1 treated with control (scramble) siRNA or BAD-LAMP siRNA and then stimulated with fluorescent CpG A for indicated times. Imaging of VAMP3 and CpG (top) or LAMP1, LAMP2 and CpGA (bottom) was performed.

Pictures are representative of n=3 experiments. Arrows indicate co-localization area. Scale bars = 5µm.E. Same as D, Imaging of VAMP. (Right) Quantification of VAMP3 and LAMP2 co-localisation at different time during CpG A stimulation was performed in mock transfected cells (black line as reference), upon BAD-LAMP silencing (red line). Graphics represent Pearson's coefficient means of 50 different cells +/- SD from n>=3 independent experiments. F. Levels of phosphorylation of TBK1 as MFI from FACS intracellular staining on CAL-1 electroporated with si RNA scramble (black line) or si RNA targeting BADLAMP (red line) upon CpG A treatment. Data +/- SD from 2 independent experiments. * $P < 0.05$ by unpaired Student's T test.

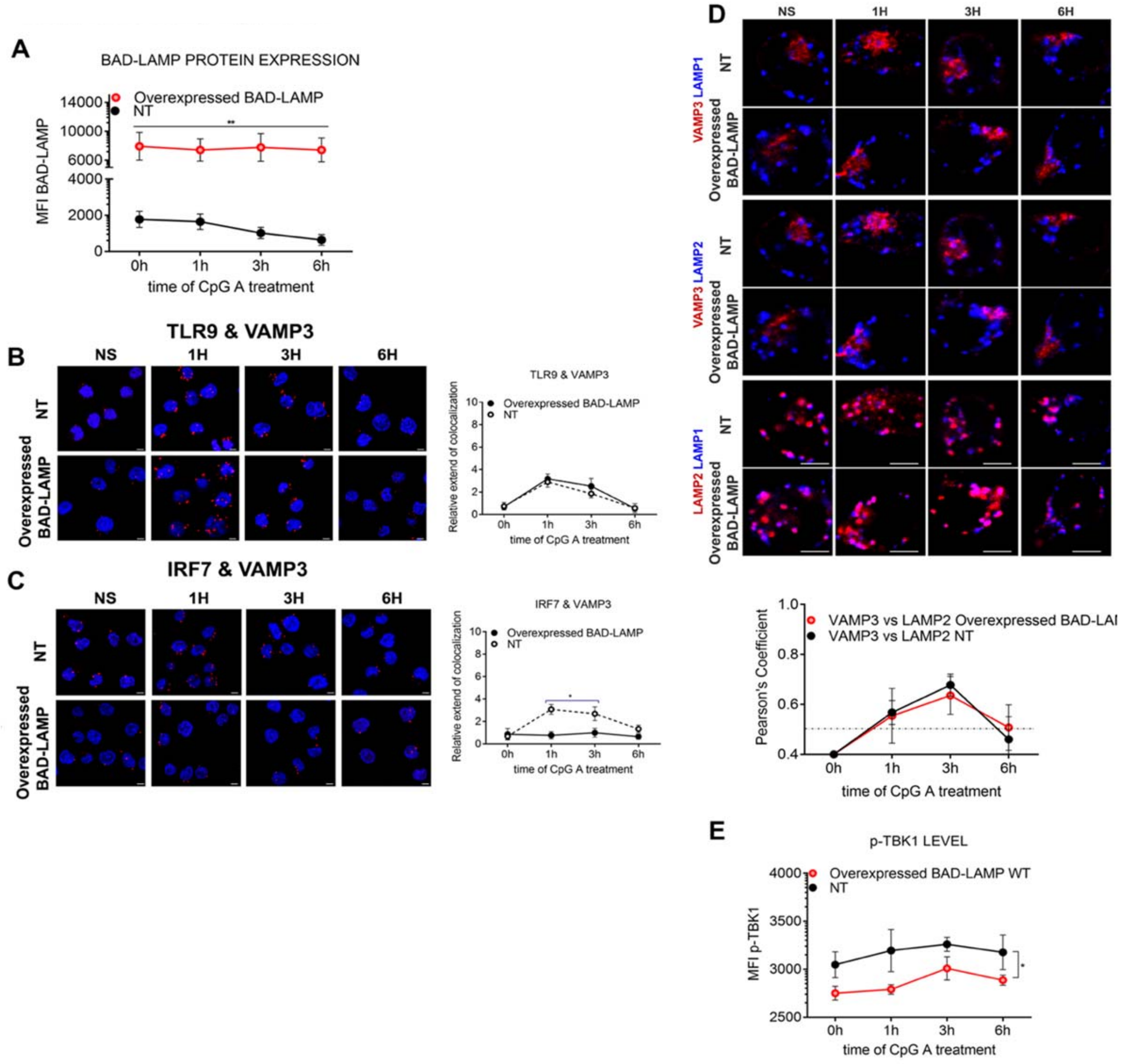

Supplementary Figure 6: BAD-LAMP over-expression prevents IRF7 recruitment on TLR9 VAMP3-positive endosomes.

A. CAL-1 cells were electroporated with BAD-LAMP mRNA or not (NT) during 6 hours prior stimulation for indicated times with CpG A. BAD-LAMP protein levels as MFI were monitored by intracellular FACS staining. Graphic represents means of MFI +/- SD from at least 3 independent experiments. B, C. (Left) Immunofluorescence proximity ligation assay (iPLA) on CAL-1 non electroporated (NT) or electroporated with BAD-LAMP mRNA and then stimulated with CpG A for indicated times. Imaging TLR9 and VAMP3 (B) IRF7 and VAMP3 (C) are presented. Confocal images are representative of 2 independent experiments where the nucleus is stained by DAPI (blue) and proximity between proteins of interest is revealed by incorporation of labelled nucleotide during PCR reaction (red). Scale bars = 5µm. (Right) Quantification of iPLA by counting the number of red dots normalized to nucleus numbers using imageJ. Graphics are representing means normalized value of 100 different cells +/- SD from at least 3 independent experiments (dots/cell). D.Left)- Immunofluorescence confocal microscopy on CAL-1 electroporated with BAD-LAMP mRNA and stimulated with fluorescent CpG A for indicated times. Images of VAMP3, LAMP1 and LAMP2 are shown and are representative of 3 independent experiments. Scale bars = 5µm. (Right) Quantification of VAMP3 and LAMP2 co-localisation at different time during CpG A stimulation was performed in mock transfected cells (black line as reference), upon BAD-LAMP mRNA electroporation (red line). Graphics represent Pearson's coefficient means of 50 different cells +/- SD from at least 3 independent experiments. E. Levels of TBK1 phosphorylation as MFI from FACS intracellular staining on CAL-1 non electroporated (black line) or electroporated (red line) with BAD-LAMP mRNA upon CpG A treatment. Data +/- SD from 2 independent experiments. * indicates that p-value are below 0,05 (Student's T test).

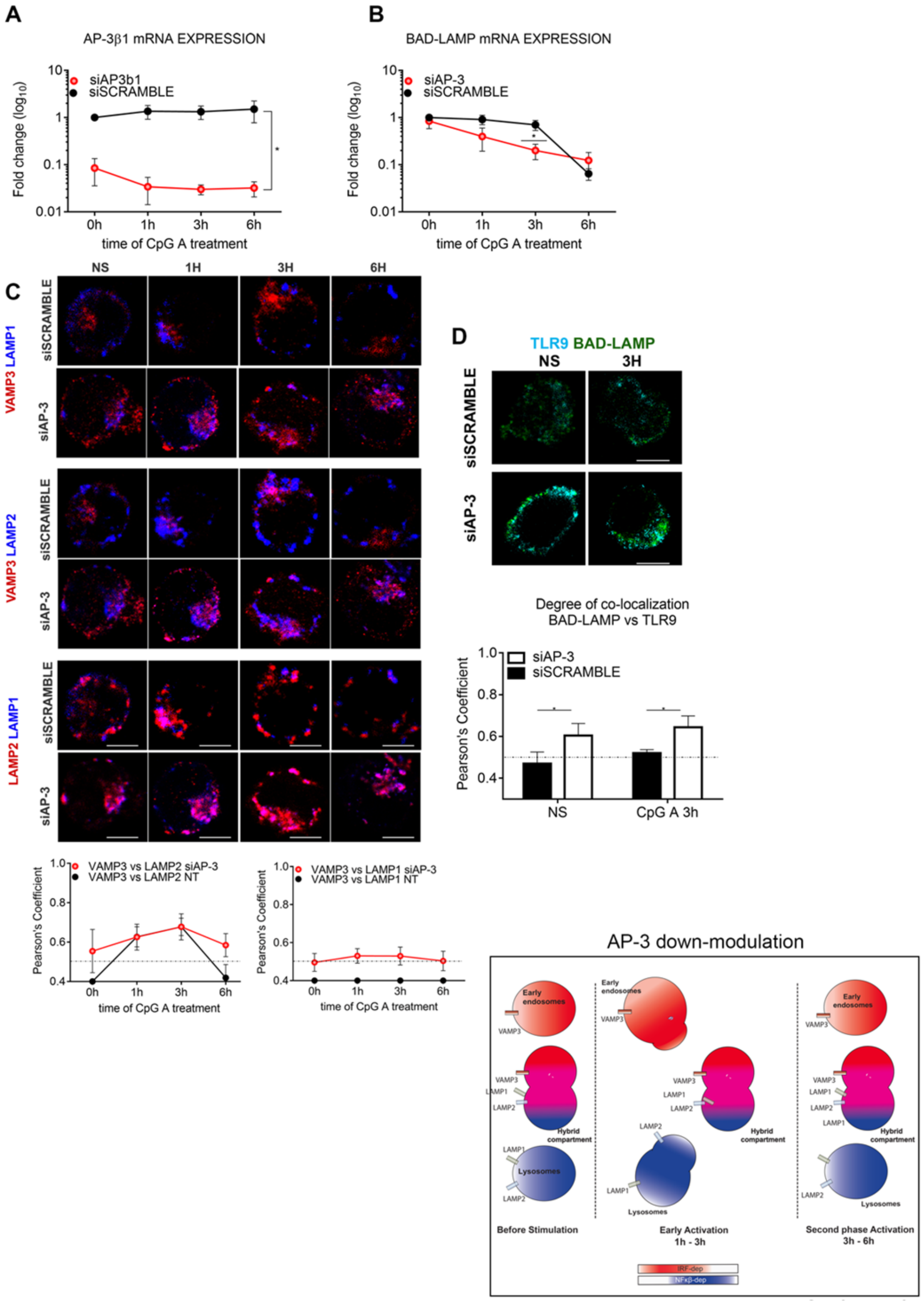

Supplementary Figure 7: AP-3 controls endosomes dynamic and TLR9-BAD-LAMP intracellular transport.

A. B. CAL-1 cells were electroporated with AP-3 siRNA or SCRAMBLE siRNA for 24hr prior stimulation during indicated times with CpG A. AP-3β1 (A) and BAD-LAMP (B) mRNA level were monitored by RT-QPCR. Raw data have been normalized to housekeeping gene (GAPDH) and graphics represent fold change +/- SD compared to non-stimulated cells from 3 independent experiments. C. D. Immunofluorescence confocal microscopy on CAL-1 electroporated with SCRAMBLE siRNA, AP-3β1 siRNA and then stimulated with CpG A for indicated times. Imaging of VAMP3, LAMP1 and LAMP2 (C) and TLR9 and BAD-LAMP (D) localization is shown. Arrows identify co-localization area. *Scale bars = 5μm.* Quantifications are shown at the bottom of the images. Graphic represents Pearson's coefficient mean for 50 different cells +/- SD from at least 3 independent experiments. A graphical abstract summarizing our results on the spatio-temporal endosomal organization in AP-3-silenced CAL-1 cells is shown. * indicates that p-value are below 0,05 (Student's T test).

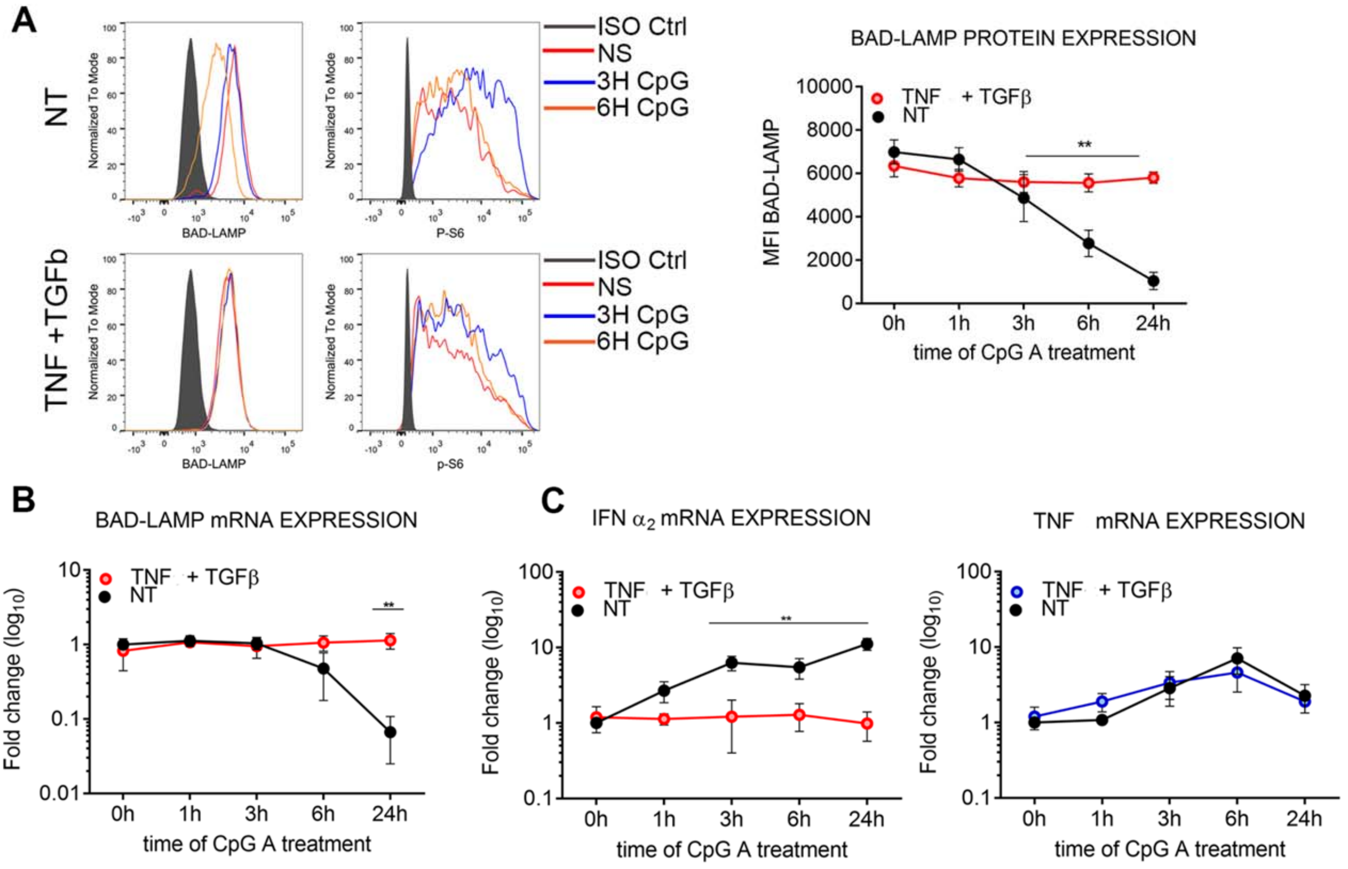

Supplementary Figure 8: BAD-LAMP expression correlates with low IFN expression in TGF-β-treated CAL-1 cells.

A. *Left)* Histogram plots from FACS intracellular staining of BAD-LAMP (left) and the phosphorylated form of S6 ribosomal protein (use as control of activation, right) in CAL-1 at steady state (red line), 3h (blue line), 6h (orange line) after CpG A stimulation. Pre-treatment for 16h with TNF-α and TGF-β was used to mimic breast tumor microenvironment inhibition on type-I IFN expression in activated pDCs (bottom panels). Full grey histograms represent isotype control. Data are representative of at least 3 independent experiments. *Right)* BAD-LAMP levels as MFI from FACS intracellular staining of CAL-1 stimulated with CpG A for indicated time with (red) or without (black) TNFα +TGF-β pre-treatment. Means +/- SD from 3 independent experiments. B. C. BAD-LAMP (B), IFN-⍰$_2$ (C left) and TNF-⍰ (C right) mRNA expression in CAL-1 stimulated for indicated time with CpG A alone (black) or with CpG A and TNF-α +TGF-β pretreatment (colored). Raw data have been normalized to housekeeping gene (GAPDH) and graphics represent fold change +/- SD compared to non-stimulated cells from 3 independent experiments. * indicates that p-value are below 0,05 (Student's T test).

Supplementary Table 1

REAGENT or RESOURCE	SOURCE	IDENTIFIER
**Antibodies**		
Anti-LAMP5 (1:100) clone 34,2	David et al 2007	N/A
Anti-LAMP5-efluor 660 (1/600) clone 34,2	Thermo Fisher Scientific	Catalog#: 50-9778-80
Anti-hTLR9 (H100) (1/50)	Santa cruz	sc-25468
Anti-UNC93B1 (E-12) (1/200)	Santa cruz	sc-135545
Anti-VAMP3 (N-12) (1/100)	Santa cruz	sc-18208
Anti-LAMP1-BV421 (1/100) clone H4A3	Biolegend	Cat#328626
Anti-STX6 (1/200)	BD Transduction Laboratories	#610636
Anti-LAMP2-AF647 (1/200) clone H4B4	Thermo Fisher Scientific	Cat#A15464
Anti-human MYD88 (1/50)	R&D systems	Cat#AF2928
Anti-S6 total (5G10) (1/1000)	Cell signalling	#2217
Anti-pS6 (ser235/236) (D57.2.2E)	Cell signalling	#5364
Anti-IRF7total (1/200) (G-8)	Santa cruz	sc74472
Anti-pIRF7 (Ser477) (1/100)	Cell signalling	#12390
Anti-AKT total (1/1000)	Cell signalling	#9272
Anti-pAKT (1/500)	Cell signalling	#4060
Anti-FLAG (M2) (1/500)	Sigma Aldrich	#F3165
Anti-TBK1total (1/1000)	Cell signalling	#3013
Anti-pTBK1 (ser172) (1/1000)	Cell signalling	#5483
Anti-P65 total (NfkB subunit) (C-20)	Santa cruz	sc-372
Anti-pP65 (NfkB subunit) (Ser536)	Cell signalling	#3033
Anti STAT1 total	Cell signalling	#9172
Anti-pSTAT1 (Y701) (58D6)	Cell signalling	#9176
Anti βactin (AC74)	Sigma Aldrich	#A2228
Anti BDCA4-PECY7 (1/500) (12C2)	Biolegend	#354507
Anti BDCA2-APC (1/500) (201A)	Biolegend	#354205
Anti-CD123-efluor450 (1/100) (6H6)	Thermo Fisher Scientific	#48-1239-42
Anti-HLA-DR PECY5 (1/100) (L243)	Biolegend	#307607
Anti-IFNa-PE (1/10) (LT27:295)	Miltenyi Biotec	#130-099-233
Anti-TNFa-Alexa700 (1/100) (MAb11)	Thermo Fisher Scientific	#56-7349-42
Anti-hIFNAR chain 2 (5ug/mL)	PBL	#21385-1
**Bacterial Strain**		
Top 10 chemically competent E.Coli	Thermo Fisher Scientific	#C404010
HST08 chemically competent bacteria	Takara	#636763
**Recombinant Proteins, Chemical Compounds**		
Human rIFNβ (1U/mL)	PBL	#11410-2
Human rTGFβ (10ng/mL)	R&D systems	#7754-BH/CF
Human rIL-3 (1ng/mL)	R&D systems	#AAC08706
Human rTNFα (1ng/mL)	R&D systems	#210-TA/CF
Bafilomycin A1 (200uM)	VWR	#J61835.MX
Z-FA-FMK (10uM)	Sigma Aldrich	#C1480, Park et al 2008
CHLOROQUINE DIPHOSPHATE CRYSTALLINE (50uM)	Sigma Aldrich	#C6628
Tumor supernatant	Sisirak et al 2013	N/A
ODN 2216 (CpG A) (5'-ggGGGACGA:TCGTCgggggg-3)	Tebu-bio	N/A

Supplementary Table 2.

**Critical Commercial Assays**		
hTNFa Elisa kit	Thermo Fisher Scientific	#88734676
Verikine hIFNα ELISA kit (all subtypes)	PBL	#41410, Steinhagen et al 2012.
mMESSAGE mMACHINE T7 kit	Thermo Fisher Scientific	#AM1344
Poly-A Tailling kit	Thermo Fisher Scientific	#AM1350
Infusion cloning kit	Takara	#638909
Amaxa Kit V Nucleofector kit	Lonza	#VACA-1003
Plasmacytoid dendritic cell kit II negative purification	Miltenyi Biotec	130-097-415, Sisirak et al 2013
RNeasy mini kit	QIAGEN	#74104
Rnase free DNAse set	QIAGEN	#79254
Superscript II for RT	Thermo Fisher Scientific	#18064014
Sybr green Premix ex TAQ II	Takara	#RR820A
PLA anti mouse probe minus (Duolink)	Sigma Aldrich	#DUO92004
PLA anti Rabbit probe plus (Duolink)	Sigma Aldrich	#DUO92002
PLA anti-Goat probe minus (Duolink)	Sigma Aldrich	#DUO92006
PLA wash buffers (Duolink)	Sigma Aldrich	#DUO82049
PLA probe maker minus (Duolink)	Sigma Aldrich	#DUO92010
PLA detecion orange (Duolink)	Sigma Aldrich	# DUO92007
**Recombinant DNA**		
pGEM4Z-FLAG-hBAD-LAMP (LAMP5)	This paper	N/A
pGEM4Z-FLAG-hBAD-LAMP Y276A (LAMP5)	This paper	N/A
**Software**		
ImageJ and JACOP plugin	Rasband, W.S., ImageJ, U. S. NIH, USA	https://imagej.nih.gov/ij/, 1997-2014)
Imaris and coloc tool	BITPLANE	http://www.bitplane.com/imaris
Flowjo	flowjo,LLC	https://www.flowjo.com/solutions/flowjo
LAS AF	LEICA	http://www.leica-microsystems.com
Zen	ZEISS	https://www.zeiss.fr/
7500 QPCR analyser software	Thermo Fisher Scientific	https://www.thermofisher.com/fr/
Graphpad prism	GraphPad Software	https://www.graphpad.com/scientific-software/prism/
**Experimental model: Cell line**		
CAL-1 cell line	Gift from Dr Maeda	Maeda et al. *International journal of hematology* 2005

Supplementary Table 3.

Oligonucleotides		
hBAD-LAMP	Defays et al 2011	N/A
Fwd : 5' GTGGCTTGGCTTTGACTCTC 3'		
Rev : 5' AGGGGTTTCCTCAAAGA 3'		
GAPDH	This paper	N/A
Fwd : 5' CAGCCTCAAGATCATCATCAGCA3'		
Rev : 5'TGTGGTCATGAGTCCTTCCA 3'		
OAS1	This paper	N/A
Fwd : 5' GTCTTCCTCAGTCCTCTCTCAC 3'		
Rev : 5' CTCTCTCTTGACAGCTTC 3'		
IFNα^{2}	This paper	N/A
Fwd : 5' TGATCCAGCAGATCTTCAAT 3'		
Rev : 5' CAGCTGCTGGTAGAGTTCA 3'		
IFNβ^{1}	This paper	N/A
Fwd : 5' TGCTTGGATTCCTACAAAGA 3'		
Rev : 5' GGATGTCAAAGTTCATCCTG 3'		
TNFα	This paper	N/A
Fwd : 5' CCCTCAGCAAGGACAGCAGA 3'		
Rev : 5' AGCCGAGGGTCAGTATGTGAG 3'		
AP-2μ1	This paper	N/A
Fwd : 5' CAAAGGCACAGCTGATGAAA 3'		
Rev : 5' GTCCCACTTCTCGCACTAGC 3'		
AP-3β1	This paper	N/A
Fwd : 5' TTTTACCTGTGGCCATGTCA 3'		
Rev : 5' AGGGACTGCACCTACATTGG 3'		
siRNA against LAMP5 (Flexitubes)	QIAGEN	#GS24141
siRNA against UNC93B1 (Flexitubes)	QIAGEN	#GS81622
siRNA against AP-3b1	QIAGEN	#GS8546

Supplementary Figure 9: Raw blots related to Fig. 1D

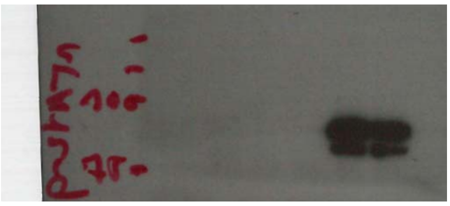

pSTAT1 84-91kDa

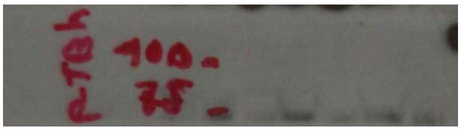

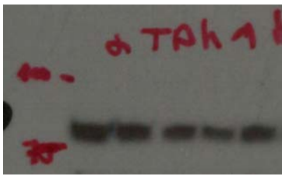

TBK1total

75kDa

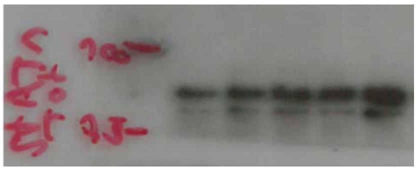

STAT1 total 84-91kDa

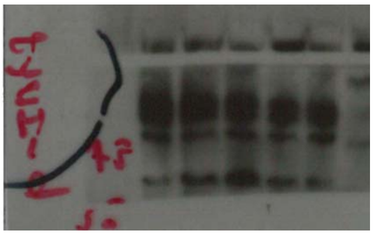

pIRF7

65kDa

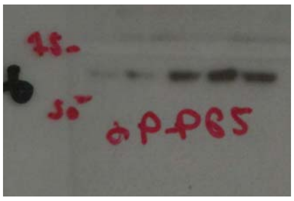

p-P65

65kDa

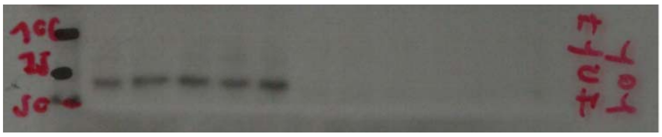

IRF7total

65kDa

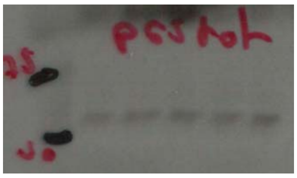

P65 total

65kDa

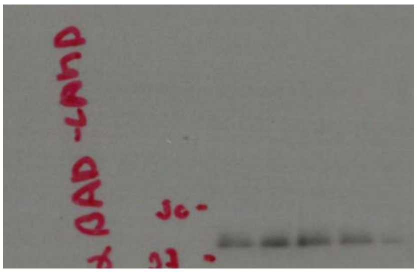

BAD-LAMP

37kDa

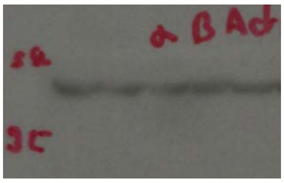

βactin

42kDa

Supplementary Figure 10: Raw blots related to Fig. 1E

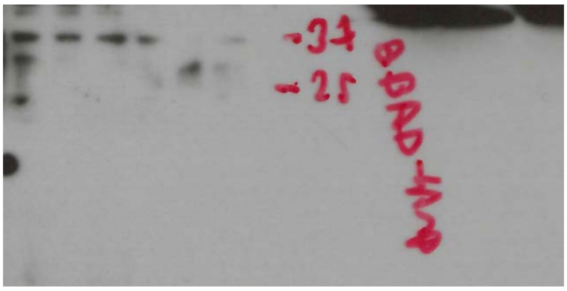

BAD-LAMP 37kDa

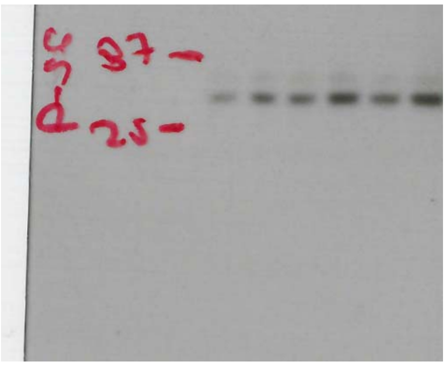

p-S6
32kDa

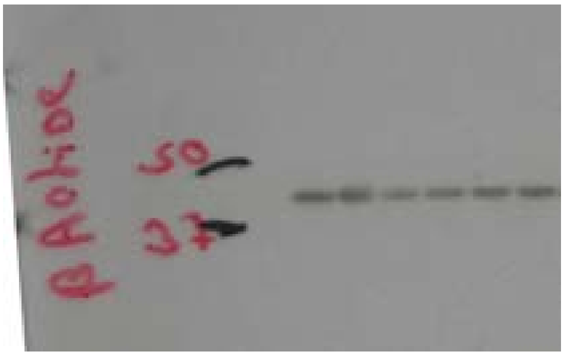

β–actin 42kDa

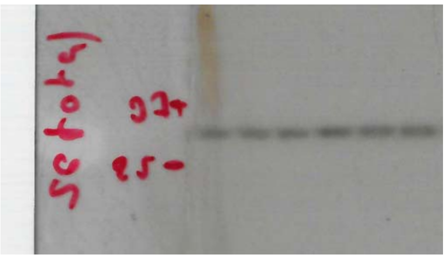

S6 total
32kDa

Supplementary Figure 11: Raw blots related to Supplementary Figure 2E

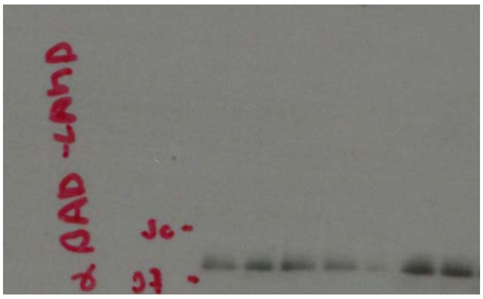

BAD-LAMP 37kDa

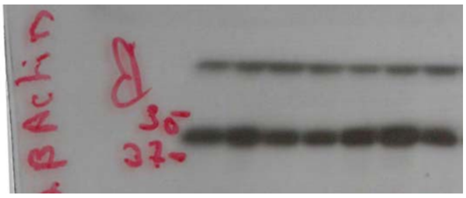

β-actin 42kDa

Supplementary Figure 12: Raw blots related to Fig. 5D

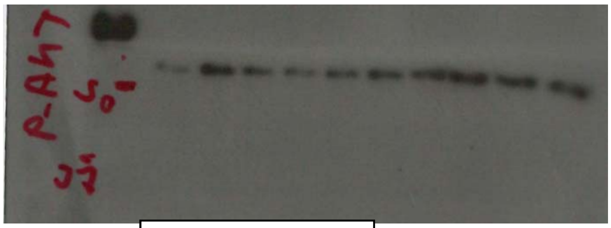

p-AKT 60kDa

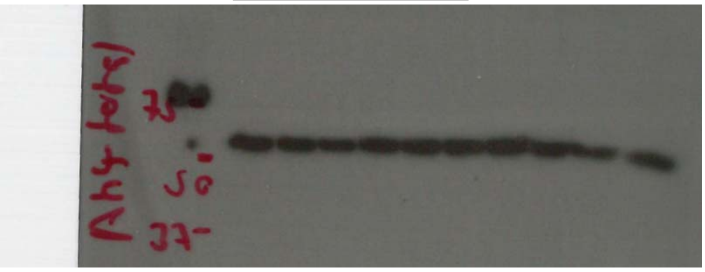

AKT total 60kDa

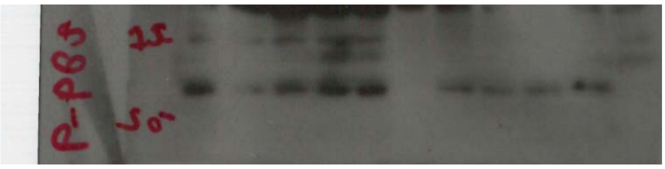

p-P65 65kDa

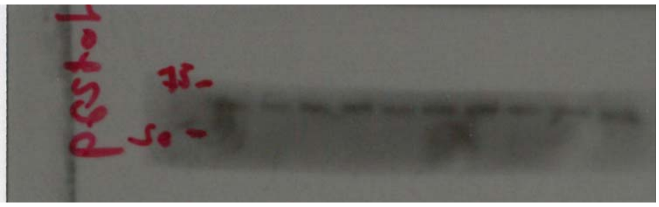

P65 total 65kDa

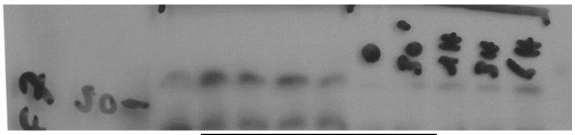

p-IRF7 65kDa

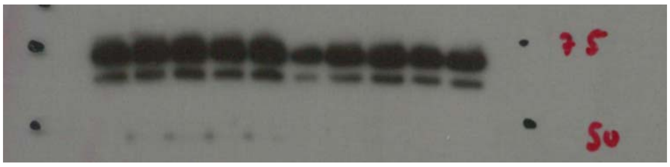

IRF7 total 65kDa

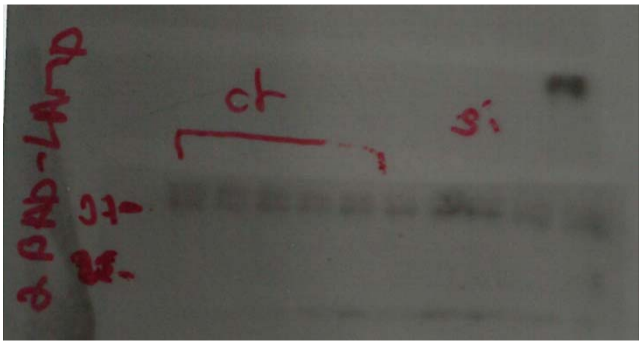

BAD-LAMP 37kDa

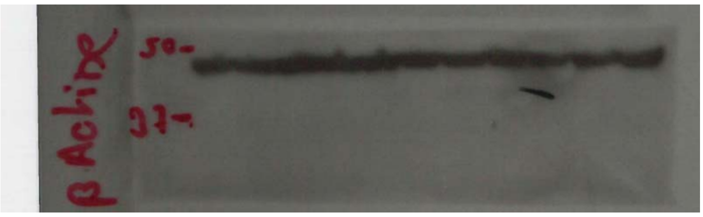

β–actin 42kDa

Supplementary Figure 13: Raw blots related to Fig. 6F

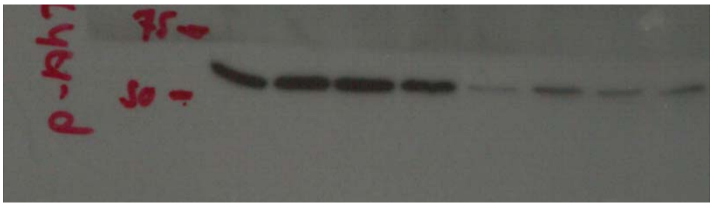

p-AKT 60kDa

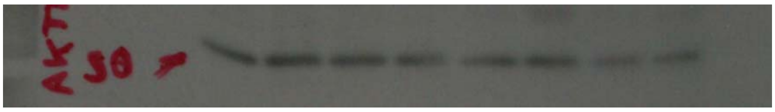

AKT total 60kDa

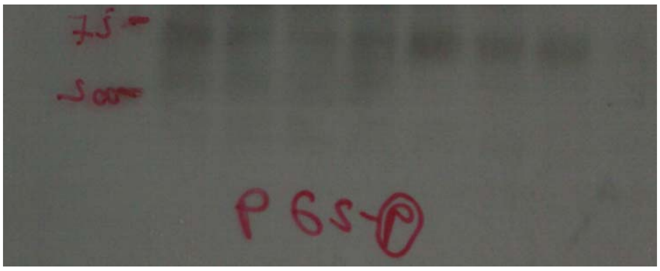

p-P65 65kDa

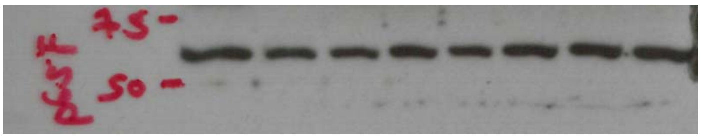

P65 total 65kDa

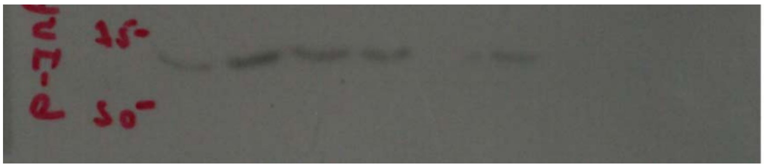

p-IRF7 65kDa

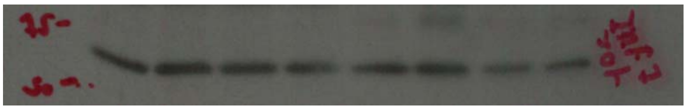

IRF7 total 65kDa

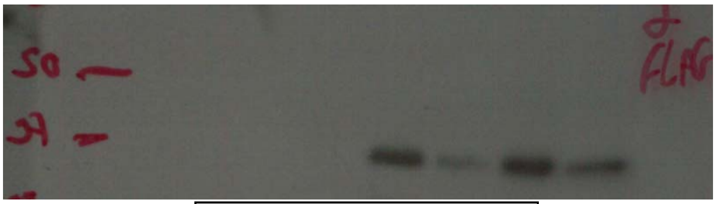

FLAG 37kDa

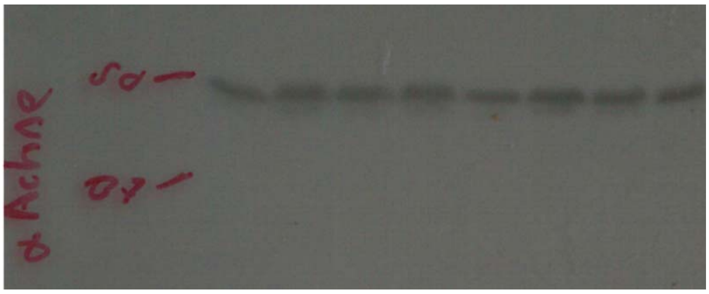

β-actin 42kDa
